# Genomic crossroads between non-Hodgkin’s lymphoma and common variable immunodeficiency

**DOI:** 10.3389/fimmu.2022.937872

**Published:** 2022-08-05

**Authors:** Kissy Guevara-Hoyer, Jesús Fuentes-Antrás, Eduardo de la Fuente-Muñoz, Miguel Fernández-Arquero, Fernando Solano, Pedro Pérez-Segura, Esmeralda Neves, Alberto Ocaña, Rebeca Pérez de Diego, Silvia Sánchez-Ramón

**Affiliations:** ^1^ Cancer Immunomonitoring and Immuno-Mediated Pathologies Support Unit, IdSSC, Department of Clinical Immunology, San Carlos Clinical Hospital, Madrid, Spain; ^2^ Department of Clinical Immunology, IML and IdSSC, San Carlos Clinical Hospital, Madrid, Spain; ^3^ Department of Immunology, Ophthalmology and ENT, School of Medicine, Complutense University, Madrid, Spain; ^4^ Oncology Department, San Carlos Clinical Hospital, Madrid, Spain; ^5^ Experimental Therapeutics and Translational Oncology Unit, Medical Oncology Department, San Carlos University Hospital, Madrid, Spain; ^6^ Department of Hematology, General University Hospital Nuestra Señora del Prado, Talavera de la Reina, Spain; ^7^ Department of Immunology, Centro Hospitalar e Universitário do Porto, Porto, Portugal; ^8^ Unit for Multidisciplinary Research in Biomedicine (UMIB), Hospital and University Center of Porto, Porto, Portugal; ^9^ Laboratory of Immunogenetics of Human Diseases, IdiPAZ Institute for Health Research, Madrid, Spain

**Keywords:** CVID, non-Hodgkin’s lymphoma, genomic, *in silico*, malignancy

## Abstract

Common variable immunodeficiency (CVID) represents the largest group of primary immunodeficiencies that may manifest with infections, inflammation, autoimmunity, and cancer, mainly B-cell non-Hodgkin’s lymphoma (NHL). Indeed, NHL may result from chronic or recurrent infections and has, therefore, been recognized as a clinical phenotype of CVID, although rare. The more one delves into the mechanisms involved in CVID and cancer, the stronger the idea that both pathologies can be a reflection of the same primer events observed from different angles. The potential effects of germline variants on specific somatic modifications in malignancies suggest that it might be possible to anticipate critical events during tumor development. In the same way, a somatic alteration in NHL could be conditioning a similar response at the transcriptional level in the shared signaling pathways with genetic germline alterations in CVID. We aimed to explore the genomic substrate shared between these entities to better characterize the CVID phenotype immunodeficiency in NHL. By means of an *in-silico* approach, we interrogated the large, publicly available datasets contained in cBioPortal for the presence of genes associated with genetic pathogenic variants in a panel of 50 genes recurrently altered in CVID and previously described as causative or disease-modifying. We found that 323 (25%) of the 1,309 NHL samples available for analysis harbored variants of the CVID spectrum, with the most recurrent alteration presented in NHL occurring in PIK3CD (6%) and STAT3 (4%). Pathway analysis of common gene alterations showed enrichment in inflammatory, immune surveillance, and defective DNA repair mechanisms similar to those affected in CVID, with PIK3R1 appearing as a central node in the protein interaction network. The co-occurrence of gene alterations was a frequent phenomenon. This study represents an attempt to identify common genomic grounds between CVID and NHL. Further prospective studies are required to better know the role of genetic variants associated with CVID and their reflection on the somatic pathogenic variants responsible for cancer, as well as to characterize the CVID-like phenotype in NHL, with the potential to influence early CVID detection and therapeutic management.

## 1 Introduction

Common variable immunodeficiency (CVID) is the most prevalent symptomatic primary immunodeficiency (PID) and is characterized by an increased predisposition to recurrent infections resulting from the low production of antibodies against pathogens (1). CVID also entails an increased risk of autoimmune, inflammatory, and malignant diseases (2). Malignant neoplasms are a leading cause of death in CVID patients and may be the first clinical manifestation of the disease (3–5). Hematologic and gastric cancers are the most frequent, with an estimated increased risk of 10- to 47-fold with respect to the general population (6–8). The incidence of cancer diagnoses does not seem to be age-dependent, and the identification of robust clinical predictors and diagnostic biomarkers represents an unmet need for CVID patients (9).

Multiple carcinogenetic mechanisms have been proposed to operate in CVID. They can be classified as cell-intrinsic mechanisms, encompassing defects of DNA repair, T–B co-stimulation, immunoglobulin gene recombination (VDJ), class-switch recombination, and somatic hypermutation (SHM); and cell-extrinsic mechanisms, including inadequate immune responses that facilitate chronic infections, typically caused by EBV, HPV, and *Helicobacter pylori* (1, 10–13), defective immune surveillance against tumors, and dysbiosis and chronic inflammation, among others (13–16). To date, only a high level of clinical suspicion enables the early diagnosis of cancer in CVID patients (4, 9, 17–20). Conversely, patients with a new diagnosis of malignancy are not routinely screened for a potential underlying CVID (17, 21). Although fortunately low, the overall incidence of malignancies in CVID patients has increased in the last decades (9, 17, 22).

We have explored in this work the genetic crossovers between CVID and NHL at the somatic level, notwithstanding that a proportion of somatic variants may underlie at the germline level and may condition the accumulation of mutagenic variants in NHL. The two conditions are epidemiologically related in the direction of CVID toward NHL. Indeed, an extensive meta-analysis by Kiaee et al., deciphering the landscape of malignancy within CVID, encompassed 48 studies worldwide with a total cohort of 8,123 CIVD patients, of which 790 cases were associated with malignancy. NHL stood out with the highest prevalence (41%) in patients with CVID with associated malignancy (4). It should be noted that despite knowing this high predominance, the mechanisms that inter non-synonymous mutations in PIK3CD, twine these two pathologies remain an enigma (23–25). The association the other way around (NHL towards CVID) remains unknown. In NHL, B cells are the subject and the target of the disease, and indeed, a variable degree of immunodeficiency both in clonal and non-clonal cells is observed. We sought to determine as a working hypothesis that there must be gene networks associating CVID and lymphoma.

NHL entails a heterogeneous group of lymphocytic disorders ranging in aggressiveness from very indolent cellular proliferation to highly aggressive and rapidly proliferative processes (26). Even though it is true that several genes and pathways are involved in the development of lymphoma, not all of them are necessarily involved in the associated immunodeficiency. Indeed, there is a diverse clinical and immunological profile in NHL patients, which will determine different predispositions to recurrent infections and the need of starting replacement therapy with immunoglobulins to manage infectious diseases (26–28).

A single and specific defect is not known to cause CVID. To date, the multiple clinical and immunological features derived from the disease are not attributed, as in other PIDs, to the alteration of a single gene expression. CVID appears to be the result of several factors contributing to a defect in antibody production, where genetic, epigenetic, and environmental factors are involved (1, 21, 23, 29, 30). It has been described that around 20% of CVIDs are associated with a monogenic defect, so approximately the remaining 80% might account for a not yet identified gene or to the combined effects of digenic or oligogenic lesions (probably common and rare prevalence ones) and triggered by external factors (23, 31–33).

On the other hand, it should not be forgotten that there is a major pool of patients initially diagnosed with “CVID” who, after performing the genetic study, present a pathogenic variant of the established monogenic IEI (34–41), such as the case of NFKB1 haploinsufficiency or activated PI3K delta syndrome (described below). These patients are classified as “CVID phenotype” by the IUIS (40, 42), although they really could encompass a more complex genetic scenario (2, 43, 44). In the face of these findings, the differential diagnosis of both pathologies should always be examined.

The current challenge encompasses identifying the bases of the germline predisposition variants present in CVID and their role in cancer development. There is increasing evidence of how germline pathogenic variants can act as an oncogenic modifier, thus determining complimentary somatic variants necessary for the development of malignancy, as well as how these variants can behave as co-oncogenes through interactions with existing somatic pathogenic variants, conditioning tumorigenesis (45–49). The identification of molecular predictors of malignancy in CVID could enable the implementation of precision medicine in this population and substantially impact follow-up strategies and treatment decisions (21, 50). However, the design of adequately powered genome-wide studies in CVID is hindered by the limited casuistry and high costs of untargeted sequencing. The combination of publicly available genomic repositories and web tools for enrichment analyses may facilitate hypothesis generation.

We have undertaken an approximation between possible genetic interactions that justify a greater predisposition of this population to the development of malignancy. For this purpose, we performed an *in-silico* analysis to identify potential common genomic substrates of CVID and NHL that can favor the design of prospective studies with clinically and immunologically annotated cohorts.

## 2 Methods

### 2.1 Overall study design

This is a non-interventional study based on the analysis of real-world data contained in cBioPortal and using web-based analytical tools. The study was carried out in the Cancer Immunomonitoring and Immuno-Mediated Pathologies Support Unit of the Clinical Immunology Department, in close collaboration with the Oncology Department, San Carlos Clinical Hospital (HCSC), in Madrid, Spain.

### 2.2 Data collection and processing

#### 2.2.1 Gene alterations associated with CVID

A systematic search of electronic databases was conducted to identify gene alterations recurrently associated with CVID following the Preferred Reporting Items for Systematic Reviews and Meta-Analyses (PRISMA) guidelines (**Supplementary Figure 1**). English-language articles published in peer-reviewed journals and conference abstracts from 1979 to 30 August 2021 were identified in Medline, EMBASE, Cochrane Central Register of Controlled Trials (CENTRAL), and Cochrane Database of Systematic Reviews, using the terms “Common Variable” and “Gene” and excluding preclinical studies. Two investigators (KG-H, JF-A) extracted data from the included studies and a third investigator (SS-R) decided on unclear or conflicting data. The titles and abstracts were evaluated, and potentially relevant publications were retrieved in full. Overall, 3,420 articles were obtained from the database and manual searches, of which 311 articles were reviewed in full. A total of 50 of the most frequent genes associated with CVID with the main described pathogenic variants were selected for analysis (**Supplementary Table 1**).

#### 2.2.2 Datasets, gene ontology, and functional analysis

We used data contained at cBioPortal (https://www.cbioportal.org, accessed in September 2021) from six studies in NHL (*n* = 1,309) to explore the distribution of genes with somatic variants occurring in the main CVID-associated germline genes associated with the pathogenic variant set. The pathogenic variants related to CVID and CVID phenotype found in the altered genes in NHL were verified through bibliographic sources as well as consulted in the ClinVar-NCBI tool (https://www.ncbi.nlm.nih.gov/clinvar/) (**Supplementary Table 2**). Genes such as *KMT2C* or the MSH family, despite not presenting clear CVID-associated pathological variables described in the literature, have been depicted as possibly harmful with a high frequency of heterozygotes compared with controls. These genes play an essential role in the associated CVID pathophysiology, such as somatic hypermutation or germline variation in cancer-susceptibility genes (51–54), for which they were considered in the analysis. Data from the VAF of the somatic pathogenic variants analyzed could not be fully retrieved. The biological functions of each gene were obtained using the 2018 Molecular_function Gene Ontology Terms through the publicly available EnrichR online platform (https://maayanlab.cloud/Enrichr/, accessed on 8 September 2021).

We used the online tool STRING (http://www.string-db.org) to construct interactome maps of genes of interest. The closer the local clustering coefficient is to 1, the more likely it is for the network to form clusters. The PPI enrichment *p*-value indicates statistical significance. Proteins are considered hubs when they have more interactions than the average. Co-occurrence analysis for gene alterations was evaluated using the cBioPortal online platform (http://www.cbioportal.org, accessed on 8 September 2021) (**Supplementary Table 3**).

This tool calculates the odds ratio (OR) for each pair of query genes, indicating the likelihood that the alterations for the two genes are co-occurrent in the selected cases, by the application of a Fisher’s exact test (statistical significance *p* < 0.05). Data analysis was performed using R (version 1.3.1093). The interaction network was mapped as default by the STRING database analysis.

## 3 Results

### 3.1 Study population

The six NHL datasets (**Table 1**) (45, 56–60) combined accounted for a total of 1,309 samples. The distribution according to sex was 50.8% men and 40.4% women (8.8% not specified). The mean age ranged from 65 to 70 years old, distributed from a minimum of 3 to a maximum of 93 years of age. The age of diagnosis was not available for analysis.

**Table 1:** NHL datasets available in cBioPortal (www.cbioportal.org; accessed in September 2021).

NHL-associated studies	Reference	CVID genes altered per study
Diffuse large B-cell lymphoma	DFCI, Nat Med 2018 (55)	32.85% (44/135 cases)
Diffuse large B-cell lymphoma	Broad, PNAS 2012 (56)	29.31% (17/58 cases)
Diffuse large B-cell lymphoma	Duke, Cell 2017 (45)	21.38% (214/1001 cases)
Diffuse large B-cell lymphoma	TCGA, PanCancer Atlas 2018 (57)	62.5% (30/48 cases)
Diffuse large B-cell lymphoma	BCGSC, Blood 2013 (58)	26.42% (14/53 cases)
Non-Hodgkin’s lymphoma	BCGSC, Nature 2011 (59)	28.57% (4/14 cases)

### 3.2 CVID-associated gene set in NHL

The prevalence of alterations in the main 50 CVID-associated genes and their genetic variants was investigated in the NHL mutation datasets. A total of 323 from 1,309 samples (25%) harbored at least one alteration, ranging from 21.38% to 62.50% according to the series. The main genetic alterations associated with CVID found in the different studies and the proportions and numbers of altered samples are shown in **Table 2**.

**Table 2:** Main CVID-associated genes and their alteration in the NHL datasets.

Gene	Prevalence (%) of samples studied (somatic mutation)	No. of samples altered	No. of patients w/ exclusive mutation	No. of patients w/ ≥ 2 associated mutations
*PIK3CD*	6	72	49	23
*KMT2C*	5	64	45	19
*STAT3*	4	57	32	25
*MSH2*	3	40	31	9
*NFKB2*	2.4	31	18	13
*PTEN*	1.8	24	22	2
*PIK3R1*	1.3	17	10	7
*LRBA*	0.9	12	6	6
*MSH5*	0.8	10	7	3
*CLEC16A*	0.5	7	2	5
*PLCG2*	0.5	6	1	5
*IRF2BP2*	0.5	6	0	6
*RAC2*	0.5	6	0	6
*ATP6AP1*	0.5	6	1	5
*CR2*	0.4	5	2	3
*TNFRSF4*	0.4	5	0	5
*DOCK8*	0.3	4	1	3
*PRKCD*	0.3	4	2	2
*TNFRSF1A*	0.3	4	3	1
*CD19*	0.2	3	1	2
*IKZF1*	0.2	3	1	2
*VAV1*	0.2	3	3	0
*NOD2*	0.2	3	2	1
*PMS2*	0.2	3	0	3
*TNFRSF13C*	0.2	3	2	1
*SH3KBP1*	0.2	3	2	1
*ARHGEF1*	0.2	3	0	3
*CD84*	0.2	2	0	2
*IL10RA*	0.2	2	1	1
*MS4A1*	0.2	2	0	2
*NFKB1*	0.2	2	1	1
*TNFRSF11A*	0.2	2	1	1
*TNFSF11*	0.2	2	1	1
*MOGS*	0.2	2	0	2
*ACOT4*	0.1	1	0	1
*CTLA4*	0.1	1	1	0
*FCGR2A*	0.1	1	0	1
*OR10X1*	0.1	1	0	1
*SLC25A5*	0.1	1	0	1
*STXBP2*	0.1	1	1	0
*TRNT1*	0.1	1	0	1
*TNFRSF13B*	0.1	1	0	1
*TNFSF13*	0.1	1	0	1
*TNFSF12*	0.1	1	0	1

The percentage, number of altered samples (≥1), and number of patients/mutations are shown.

The prevalence of gene alterations in NHL was compared with that reported as genetic alterations in CVID (50, 61). Approximately up to 25% of patients with CVID carry a germline alteration in non-consanguineous populations (31). However, the prevalence of specific gene alterations remains largely unknown, particularly those categorized as rare functional variants and those which independently do not exhibit a causal relationship (**Table 3**) (21, 61, 62). The most prevalent gene alterations in CVID were *PIK3CD* (2.6%), *LRBA* (2.6%), and *NFKB2* (0.5%), while the most frequent in NHL were *PIK3CD* (6%), *KMT2C* (5%), and *STAT3* (4%). **Supplementary Table 2** shows the genes with pathogenic variables associated with the CVID-like phenotype and their frequency found in NHL samples.

**Table 3:** Frequencies of the main CVID-associated gene alterations in the NHL and CVID populations.

Gene symbol	Prevalence of the samples studied (somatic mutation)	CVID prevalence (germline mutation)	Gene symbol	Prevalence of the samples studied (somatic mutation)	CVID prevalence (germline mutation)
PIK3CD	6	2.674	NOD2	0.2	Unknown prevalence
KMT2C	5	Unknown prevalence	PMS2	0.2	Unknown prevalence
STAT3	4	Unknown prevalence	TNFRSF13C	0.2	0.10
MSH2	3	Unknown prevalence	SH3KBP1	0.2	Unknown prevalence
NFKB2	2.4	0.535	ARHGEF1	0.2	Unknown prevalence
PTEN	1.8	Unknown prevalence	CD84	0.2	Unknown prevalence
PIK3R1	1.3	0.481	IL10RA	0.2	Unknown prevalence
LRBA	0.9	2.674	MS4A1	0.2	0.053
MSH5	0.8	0.4	NFKB1	0.2	0.16
CLEC16A	0.5	Unknown prevalence	TNFRSF11A	0.2	Unknown prevalence
PLCG2	0.5	0.214	TNFSF11	0.2	Unknown prevalence
IRF2BP2	0.5	0.053	MOGS	0.2	Unknown prevalence
RAC2	0.5	0.053	ACOT4	0.1	Unknown prevalence
ATP6AP1	0.5	Unknown prevalence	CTLA4	0.1	0.642
CR2	0.4	0.107	FCGR2A	0.1	Unknown prevalence
TNFRSF4	0.4	Unknown prevalence	OR10X1	0.1	Unknown prevalence
DOCK8	0.3	Unknown prevalence	SLC25A5	0.1	Unknown prevalence
PRKCD	0.3	0.214	STXBP2	0.1	Unknown prevalence
TNFRSF1A	0.3	Unknown prevalence	TRNT1	0.1	Unknown prevalence
CD19	0.2	0.374	TNFRSF13B	0.1	0.07
IKZF1	0.2	0.321	TNFSF13	0.1	Unknown prevalence
VAV1	0.2	0.053	TNFSF12	0.1	0.053

### 3.3 Characterization of the functional networks, gene ontology, and biological processes of the genes altered in both NHL and CVID

**Figure 1:**
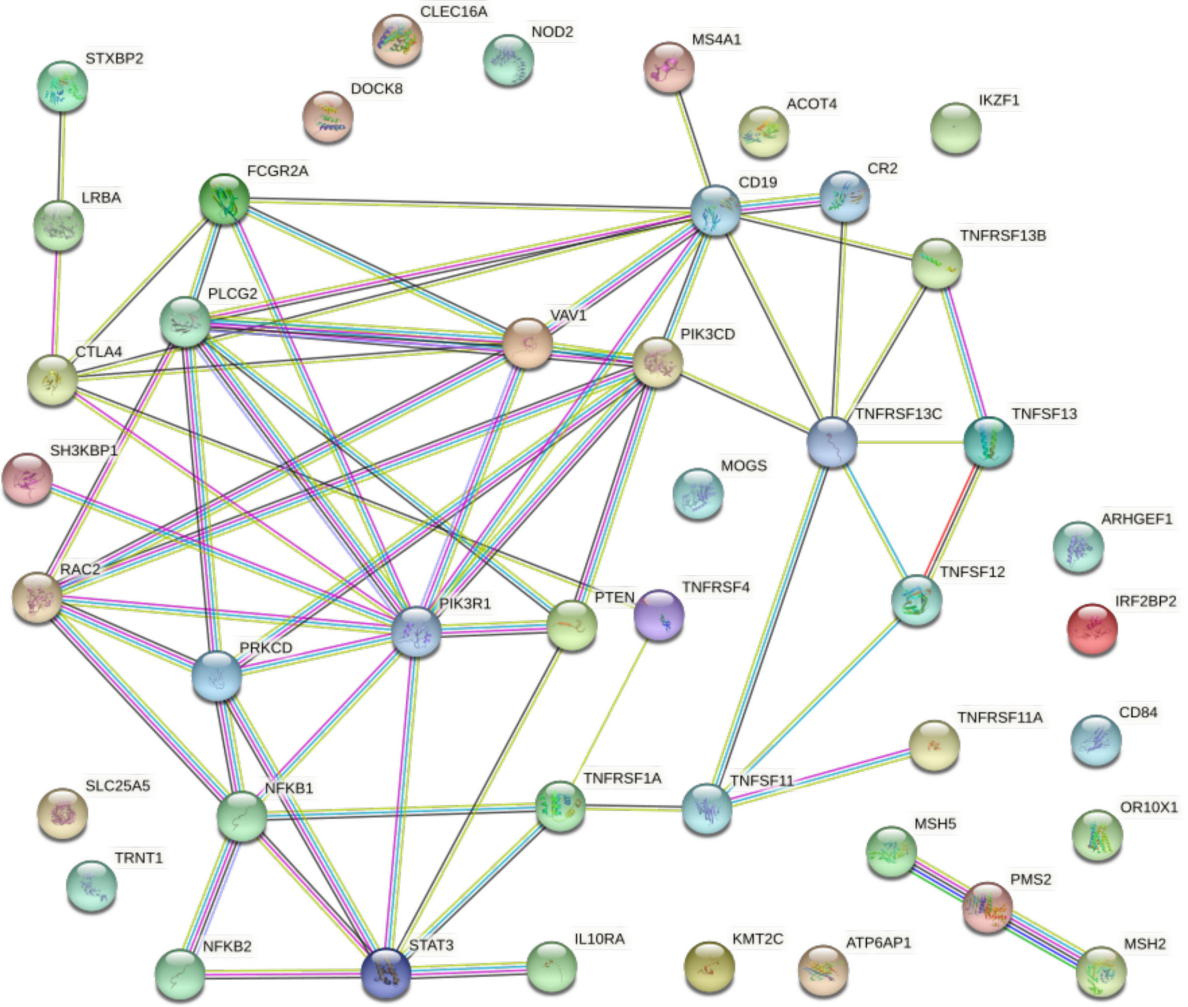
Protein–protein interaction map displaying the significant functional network integrated by the selected genes involved in NHL and CVID. Known interactions are displayed in blue and pink lines. Predictive interactions include the following: green line, gene neighborhood; red line, gene fusions; blue line, gene co-occurrence; yellow line, text mining; black line, co-expression; and gray line, protein homology. Connections are filtered by the highest confidence (0.7). PPI enrichment *p*-value: < 1.0e−16.

In order to unveil the putative functional connection between the most prevalent gene alterations in both NHL and CVID, we interrogated the STRING database and generated protein–protein interaction maps adjusted by the type and strength of the interaction (**Figure 1**). A confidence threshold of 0.7 was set between the nodes (PPI enrichment *p*-value: <1.0e−16), with 14 nodes lacking predicted interactions. Notably, PIK3R1 appeared as a central node connecting the PI3K pathway and downstream mediators with different immune mediators such as STAT3, NFKB2, CTLA4, or CD19.

To further investigate the functional characterization of the set of CVID-associated genes, we interrogated the EnrichR database and found that inflammation, immune surveillance, and defective DNA repair appeared as significantly enriched biological processes (**Table 4**), with recurrent functions in cytokine and TNF signaling.

**Table 4:** Relationships organized in biological pathways and processes mainly related to CVID-associated genes expressed in NHL (sorted by *p*-value; *p* < 0.05).

Associated pathways of CVID genes	Related genes
Antigen activates B-cell receptor (BCR) leading to the generation of second messengers	*CD19*, *PLCG2*, *PIK3CD*, *PIK3R1*
Role of phospholipids in phagocytosis	*PLCG2*, *PIK3R1*, *PRKCD*, *FCGR2A*
PKMTs methylate histone lysine	*NFKB1*, *NFKB2*, *KMT2C*
Dex/H-box helicases activate type I IFN and inflammatory cytokine production	*NFKB1*, *NFKB2*
RIP-mediated NF-κB activation *via* ZBP1	*NFKB1*, *NFKB2*
TAK1 activates NF-κB by phosphorylation and activation of the IKK complex	*NOD2*, *NFKB1*, *NFKB2*
TRAF6 mediated NF-κB activation	*NFKB1*, *NFKB2*
Mismatch repair (MMR) directed by MSH2:MSH3 (Mutsbeta)	*PMS2*, *MSH2*
Interleukin receptor SHC signaling	*PIK3CD*, *PIK3R1*
TP53 regulates the transcription of DNA repair genes	*PMS2*, *MSH2*
Interleukin-1 processing	*NFKB1*, *NFKB2*
Interleukin-10 signaling	*IL10RA*, *STAT3*, *TNFRSF1A*
IkBA variant leads to EDA-ID	*NFKB1*, *NFKB2*
TNF receptor superfamily (TNFSF) members mediating the non-canonical NF-κB pathway	*TNFSF11*, *TNFRSF11A*, *TNFRSF13C*
TNFs bind their physiological receptors	*TNFSF11*, *TNFSF13*, *TNFRSF13B*, *TNFRSF4*, *TNFRSF1A*
TNFR2 non-canonical NF-κB pathway	*TNFSF11*, *TNFSF13*, *TNFRSF13B*, *TNFRSF11A*, *TNFRSF13C*, *TNFRSF4*

### 3.4 Co-occurrence of alterations in CVID-associated genes in NHL

We next analyzed the co-occurrence of genetic alterations in the CVID-associated gene set. Sixteen pairs presented statistical significance co-occurrence (**Table 5**). Regarding the relationships organized in biological pathways and processes related to CVID-associated genes expressed in NHL, the proteins encoded by 6 of the 16 significant pairs were found to contribute jointly to a shared function. In contrast, 10 of the 16 pairs that represented independent processes did not associate with common pathways. No mutual exclusivity was found in the analysis.

**Table 5:** Co-occurrence of mutations in the analyzed population calculated by the odds ratio method in cBioPortal.

Gene A	Gene B	Neither	A not B	B not A	Both	Log2 odds ratio	*p*-value	*q*-value	Tendency	Associations with proteins encoded
*DOCK8*	*PMS2*	1,297	2	1	2	>3	<0.001	0.013	Co-occurrence	Independent roles
*CR2*	*PMS2*	1,296	3	1	2	>3	<0.001	0.013	Co-occurrence	Independent roles
*CR2*	*PTEN*	1,278	2	19	3	>3	<0.001	0.013	Co-occurrence	Independent roles
*PMS2*	*PLCG2*	1,295	1	4	2	>3	<0.001	0.013	Co-occurrence	Independent roles
*CLEC16A*	*SH3KBP1*	1,295	4	1	2	>3	<0.001	0.013	Co-occurrence	Independent roles
*CR2*	*DOCK8*	1,295	3	2	2	>3	<0.001	0.014	Co-occurrence	Independent roles
*DOCK8*	*PLCG2*	1,294	2	4	2	>3	<0.001	0.017	Co-occurrence	Shared roles
*MSH5*	*PMS2*	1,291	8	1	2	>3	<0.001	0.020	Co-occurrence	Shared roles
*CR2*	*PLCG2*	1,293	3	4	2	>3	<0.001	0.020	Co-occurrence	Shared roles
*LRBA*	*SH3KBP1*	1,289	10	1	2	>3	<0.001	0.028	Co-occurrence	Independent roles
*DOCK8*	*MSH5*	1,290	2	8	2	>3	<0.001	0.031	Co-occurrence	Independent roles
*CR2*	*MSH5*	1,289	3	8	2	>3	<0.001	0.044	Co-occurrence	Independent roles
*FCGR2A*	*OR10X1*	1,301	0	0	1	>3	<0.001	0.044	Co-occurrence	Independent roles
*TNFRSF13B*	*TNFSF13*	1,301	0	0	1	>3	<0.001	0.044	Co-occurrence	Shared roles
*TNFRSF13B*	*TNFSF12*	1,301	0	0	1	>3	<0.001	0.044	Co-occurrence	Shared roles
*TNFSF13*	*TNFSF12*	1,301	0	0	1	>3	<0.001	0.044	Co-occurrence	Shared roles

## 4 Discussion

Unraveling the molecular interplay underlying CVID is a difficult task due to the lack of consistent diagnostic protocols and the need of high clinical suspicion. Patients are often studied late after recurrent infectious episodes or associated complications including malignancy. In particular, NHL is the most prevalent malignancy in CVID patients, and its concurrence may lead to worse outcomes through either poor treatment responses and recurrent infectious complications (3, 19, 22, 24, 63). Therefore, understanding how CVID and NHL are interrelated is an unmet need that requires preliminary data to substantiate future prospective endeavors.

Similar to other malignancies, NHL B cells coordinate signaling pathways in the tumor microenvironment, promoting tumorigenesis and generating a state of immunosuppression that favors the tumor growth and progression (26, 64). B cells are the main players in both NHL and CVID, and unlike solid tumors, an alteration in both somatic and germline might condition a similar response at the transcriptional level in the shared signaling pathways, generating, in this setting, a somatic profile of immunodeficiency in NHL similar to that derived from the germline of CVID (65–67). This phenomenon may be a reflection of specific somatic pathogenic variants in the aberrant clonal B cells responsible for orchestrating key processes shared between both pathologies, such as defects in maturation and differentiation of the B lymphocyte, T–B co-stimulation, immunoglobulin gene recombination (VDJ), class-switch recombination, and somatic hypermutation (45, 52, 58, 68–71).

Pathway enrichment studies have determined that the molecular mechanisms involved in both Hodgkin’s and non-Hodgkin’s lymphoma were more enriched in those pathways associated with immune response. The vital role of pathogenesis and immune escape mechanisms and the development of an immunosuppressive tumor microenvironment have been highlighted by these analyses (64, 68, 72, 73). By way of illustration, the paths associated with the members of the Bcl-2 family, which are proteins that play an essential role in regulating cell apoptosis, survival, and proliferation, are highly expressed in most NHL (74). BCL2 is an antiapoptotic factor, and its deregulation in NHL is associated with the constitutive activation of NF-κB, which in turn influences the therapeutic response and prognosis of these patients (75, 76). On the other hand, overexpression of BCL-xL has been reported in 80% of all NHL, relating it to inhibition of cell death and, therefore, the development of malignancy (74).

Bcl‐2 and Bcl‐xl share functional homology, and their transcription is intimately regulated by NF‐κB activity (35, 77, 78). NF-κB1 loss-of-function (LoF) has been associated with an impaired function of the Bcl-2 protein in memory B cells of CVID patients, which would predispose to apoptosis and to defective differentiation and maturation of immunoglobulin-producing B lymphocytes (a typical feature of CVID) (36, 77). The latter mechanism would be contrary to what occurs in NHL, where apoptosis would be suppressed contributing to cell proliferation and malignancy (74, 75). Some series describe NF-κB as the most common monogenic cause of CVID in Europeans (34, 36). However, studies such as those by Li et al. denote that NFKB1 variants are common in healthy individuals and cannot necessarily be considered causal CVID (35).

Several signals that lead to activation of the NF-κB pathway to regulate B-cell survival in mature B cells highlighted the ligands BAFF (B-cell-activating factor of the tumor necrosis factor family) and APRIL (a proliferating inducing ligand), both joining to TACI (transmembrane activator and CAML interaction) (79). The overexpression of BAFF, APRIL, or their receptor TACI is noted in hematological malignancies such as B-cell NHL (80, 81). This would contribute to the survival and prognosis of tumor cells (37, 81, 82). TACI/APRIL function is also related to the class switch recombination of immunoglobulins (38, 39). Several variants have been considered CVID causative or modifier (83, 84), depending on the occurrence of similar variants with uncertain significance in the general population (84–87).

Since the pathways associated with tumorigenesis (such as cell death, cell proliferation, and response to stress) are shared by a large number of genes with very different or even opposite roles (69, 88), it is complicated, if not impossible, to draw a perfect line that separates the immunological pathways involved in the development of lymphoma from those of underlying immunodeficiency. The ways involved could be similar but up- or downregulated by the penetrance and expressivity of the differential genes (89–94). Our study provides first evidence of the common genomic landscape of CVID and NHL. By means of an extensive systematic search followed by an *in-silico* analysis of publicly available genomic repositories, we have identified a set of genes recurrently altered in both diseases and therefore suggestive of common functional features of immunodeficiency. Among the CVID-associated genetic variants, non-synonymous mutations in *PIK3CD*, *KMT2C*, and *STAT3* were the most prevalent in NHL. Functional enrichment analysis revealed a robust centralizing role for PIK3R1 and downstream effectors of the PI3K pathway.

*PIK3CD* exerts a key role in the activation of signaling cascades involved in cell proliferation and survival (95). Likewise, it mediates immune responses associated with the development, migration, and function of B lymphocytes and TCR signaling at the immune synapse and participates in the activation of the NK cell and in neutrophil chemotaxis, as well as in the production of cytokines in response to TLR4 and TLR9, necessary for antigenic presentation (96).

The second candidate *KMT2C* represents a catalytic subunit of the MLL2/3 coactivator complex, specific nuclear receptors for epigenetic transcriptional activation (54, 97). Pathogenic variants in *KMT2C* have been linked to leukemogenesis and disorders of cell development (98). So far, *KMT2C* does not present clear CVID-associated pathological variables described in the literature; nevertheless, this gene has been expressed as possibly harmful with a high frequency of heterozygotes compared with controls, where their function plays an essential role in the associated CVID pathophysiology such as germline variation in cancer susceptibility (49, 99).

Thirdly, an interesting finding was the prevalence of the STAT-3 gene at 4% in NHL, but unknown for CVID (63, 100). STAT-3 is a signal transducer and activator of transcription that mediates cellular responses associated with interleukins and growth factors (101). Among its multiple functions, STAT-3 intervenes in the cell cycle by inducing the expression of critical genes for the progression from G1 to S phase, is involved in the response to activated FGFR1-4 and anti-inflammatory response, and modulates differentiation toward Th17 or regulatory (T_Reg_) cells through IL-6 signaling cascade and on the promoters of several acute-phase protein genes. In a general framework, hematological malignancies associated with STAT-3 alteration are linked to gain of function (GOF) (102). STAT genes are relevant for lymphocyte survival and tumorigenesis (100–104). STAT-3 mutations are rare in B-cell NHL. However, STAT-3 GOF mutations have been associated with increased STAT-3 phosphorylation and transcriptional activity, which may foster malignant cell survival and proliferation (101). Our study detected nine pathogenic variants associated with the GOF of STAT-3 that have been described to be associated with an immunodeficiency phenotype (105, 106). In the same way, other authors have related GOF mutations in STAT-3 with several immunodeficiency phenotypes, considering it as a candidate causative gene of the CVID phenotype (63, 100).

Of note, the loss-of-function mutations in *PIK3R1* and *PTEN* lead to a similar clinical phenotype to that of GOF mutations in the *PIK3CD* gene, characterized by decreased B lymphocytes and naïve T lymphocytes, lymphoproliferation, and autoimmunity (107, 108). These genes are within our top 7 CVID-expressed genetic variants in NHL mutations and have been previously associated with lymphoma predisposition (40, 109). Pathogenic variants in the *PTEN* gene are a known substrate of the Cowden syndrome. Although atypical, its presentation as a CVID phenotype has been reported (110, 111). On the other hand, patients with pathogenic variants that activate PI3KR1 signaling have been associated with antibody deficiency partly due to the altered function of p85α (112). Low B-lymphocyte counts can also result from severely impaired B-cell development and differentiation (107). The degree of involvement is associated with the type of inheritance, the more profound being in homozygous patients (agammaglobulinemia) and variable behavior (hypogammaglobulinemia) in patients with heterozygous mutations at the *PIK3R1* splice site (107). One patient with heterozygous *PIK3R1* mutation presented the CVID phenotype, and this gene is considered causative of CVID phenotype immunodeficiency (63, 113, 114).

Patients with activated PI3Kdelta syndrome (APDS) have an increased risk of both HL and NHL. The incidence of lymphoma is as high as 13%–30% (115, 116), and a vast majority is related to EBV infection (117, 118). Although the immune escape strategies in lymphoma may vary between individuals (119), the most significant known stimuli so far related to the pathogenesis of B-cell lymphoma in APDS are related to the upregulation of the mTOR signaling pathway with the downstream effector of PI3K/AKT, increased production of transcription factors involved in the process of apoptosis, affecting the regulation of the cell cycle conducting to uncontrolled cell survival and malignant transformation (120, 121). For many years, APDS was defined as a CVID-like syndrome, especially due to its clinical similarity to that of monogenic CVID forms (107, 115, 120). However, APDS patients are more predisposed to viral infections than CVID, highlighting EBV infection (117, 122). The clinical features of cellular immunodeficiency play an essential role in APDS, such as the increased senescent T lymphocytes with shortened telomeres, especially of APDS type I (APDS1) (96, 123). The differentiation of PI3K signaling defect between both pathologies is crucial.

Our aim was to investigate the plausible common genomic grounds between CVID phenotype immunodeficiency in NHL. Therefore, an exploratory analysis of germline CVID-related gene alterations was performed in NHL tumor samples. A key immune system feature is the recombination of the (VDJ) gene segment in the generation of T- and B-cell antigen receptor (TCR and BCR) repertoire, as well as SHM of B cells in the generation of immunoglobulin isotypes (124). These processes are orchestrated and tightly regulated by the expression of specific genes, as well as their resulting proteins, which also share a function within the DNA and transcription repair mechanisms (70, 125), the alteration of the latter being widely known as cancer-predisposing factors (10, 71, 126–128).

As the availability of next-generation sequencing data increases, there is more evidence of the interrelationship of somatic and germline genetic conditions associated with immunodeficiency and cancer (46). Somatic pathogenic variants can reflect or be a consequence of alterations in germinal variants (45, 47, 65). Several studies have recently explored how germline variants can act as an oncogenic modifier, thus determining complementary somatic pathogenic variants necessary for the development of malignancy. Similarly, germline variants not directly associated with oncogenesis can behave as co-oncogenes through interactions with existing somatic pathogenic variants, thus enhancing tumorigenesis (47, 65). This phenomenon suggests that the inherited germline variants would govern where and how malignancy will develop. Germline variants of somatic alteration genes in NHL may themselves predispose to NHL in patients with CVID (3, 45, 113, 129). Since the clinical phenotype caused by germline variants can vary depending on the genetic alterations and the patient, the combination of variants could be pathogenic and lead to an increased susceptibility to developing NHL (11, 19, 58, 65, 69, 129).

The pioneer PanCanAtlas Germline Working Group has investigated the pathogenic germline variants in more than 10,000 adults within 33 types of cancer, including diffuse large B-cell lymphoma. Although the results are preliminary and the data are not yet public, the results are promising to enlighten the basis of germline predisposition variants and their role in cancer (130). Seidel et al. performed the analysis of germline variants in childhood cancers, suggesting that the development of most cancers seemed to be more related to a cell-intrinsic defect of the immune system in the detection, control, and elimination of cancer cells, rather than being directly caused by extrinsic associated factors (131). In a recent study, Hauck et al. explained how the germline genetic variants associated with IEI behave as oncogenes when they arise as somatic pathogenic variants and cause specific cancerous entities, mainly CARD11, IKZF1, GATA2, PMS2, MSH6, etc. alterations (46).

In the work of Lincoln et al., 34% of the patients (207/608) presented GPV and second neoplasms and 15% (32/207) of them carried GPV associated with specific recommendations for detection or risk reduction for their subsequent cancers (132). Given that CVID patients are at theoretical high risk for developing second neoplasms (4, 6, 10), screening of CVID-associated genes at malignancy diagnosis might modify current protocols in order to improve follow-up and prognosis.

An interesting study by Liu et al. proposed 172 possible CVID candidate genes functionally similar to known CVID genes based on their interactions and biological distance. Liu et al. studied their differential expression finding upregulation and downregulation in patients compared to controls (133). This suggests that CVID could behave as a mixed model with the presence of punctual alterations due to cumulative effects of polygenic determinants, gene–gene interactions, and/or regulatory variation in non-coding regions detrimental to a particular immune pathway rather than as an effect derived from a specific point of mutation. This overexpression could be fundamental as a complementary co-oncogene factor in the development of malignancy in CVID patients (134, 135).

Our data show that up to one-fifth of NHL patients had similar CVID-related genetic pathogenic variants in the somatic line, some with a frequency up to 6%, as is the case of *PIK3CD*. Several of these genes were significantly associated with different important pathways of the immune response, inflammation, and cell repair (45, 50, 56, 98, 136). CVID-associated genes expressed in NHL patients are also involved in DNA repair, genome integrity, immunosurveillance, and predisposition to cancer (7, 18, 113, 137). Consequently, these genes could also be described as NHL-associated GPV, as well as with other malignancies (136, 138). Interestingly, our data help to identify and prioritize those pleiotropic genes that could simultaneously affect several relevant immunological pathways. Two of the clearest examples in our study were the *NFKB1* and *NFKB2* genes that were found to be involved in pathways such as PKMT methylation of histone lysines, DEx/H-box helicase activation of type I IFN and inflammatory cytokine production, RIP-mediated NF-κB activation *via* ZBP1, and interleukin-1 processing, among others. Other examples are *MLH1*, *MSH2*, *MSH6*, and *PMS2*, genes involved in DNA damage/repair that are highly involved in immunodeficiency, NHL, constitutional mismatch repair deficiency (CMMRD), and Lynch syndrome and also associated with colorectal and endometrial cancer (129, 139, 140).

Another intriguing result of our study is the relationships among organized biological pathways and CVID-associated genetic variant processes expressed as somatic pathogenic variants in NHL, mainly the core role that *PIK3R1* seems to have as an interconnector of altered signaling pathways (**Supplementary Figure 2**). *PIK3R1* is the seventh in the gene ranking for NHL and CVID, with a prevalence of 1.3% in NHL and 0.48% in CVID (61). However, according to the predicted protein–protein interaction network, it may centralize relevant immune signaling pathways associated with immunodeficiency and malignancy (107, 112, 141). These influential hub genes interact with other nodes and thus exert a role of checkpoint alterations at the heart of both disorders (107, 112, 141).

Patients with APDS have a greater predisposition to B-cell lymphoma, for which the *PIK3R1* involvement may be considered a risk factor for carcinogenesis (107). Moreover, the spectrum of malignancy could be broader, since the involvement of p85α can also affect the function of p110α, p110β, and PTEN (141, 142), leading to hyperactivation of PI3K signaling in other cell types with a greater predisposition to malignancy (107, 141, 142).

In CVID published cohorts, the estimated prevalence of the direct mutation in *PIK3R1* is 0.481%, with its associated phenotype being *PIK3CD* which is the most frequent pathogenic variant in both CVID and NHL with the prevalence of 2.674% and 6.0%, respectively (61). In the same way, as previously mentioned, *PIK3R1* seems to be the common link among CVID-related genes in NHL, further representing a frequently associated gene alteration in many tumors (141). *PIK3R1* is altered in 3.62% of solid cancer patients and 1.52% of lymphoma patients (112, 143). In our match with NHL, the prevalence of *PIK3R1* was 1.3%, slightly lower than that described in previous studies (112, 143). Our results point to *PIK3R1* as a hotspot in the relationship between CVID and NHL, not just directly associated with its mutation, but rather with a defect in the common signaling pathways for which it is crucial (63, 96, 107, 113, 114, 141). This hotspot would not only condition the development of malignancy but also influence the immunodeficiency phenotype linked with CVID. These findings encourage further investigation on the pathogenic status and role of *PIK3R1* in CVID patients.

Our study is based on an approach to better understand the genetic link associated with signaling pathways shared between both pathologies (NHL and CVID) in a population where malignancy is a leading cause of mortality. Our preliminary data shown here align with the hypothesis that both conditions are indeed genetically bidirectionally related, and even if all the variants found were exclusively somatic variants within NHL cells, they could induce the associated immunodeficiency. It is a shame not to be able to retrieve the VAF from the somatic pathogenic variants analyzed. These data would have implications and relevance in understanding the behavior of somatic variants in malignancy as a reflection of immunodeficiency and *vice versa*.

We look forward to validating our results in independent large cohorts of immunodeficiency and cancer, as well as the detailed description of pathogenic germline variants in tumors. More evidence is required such as studies of long cohorts of immunodeficiency and cancer, as well as the detailed description of pathogenic germline variants in tumors.

Nevertheless, the continuous progress in genetic sequencing and in the availability of cancer genomic datasets opens new avenues to deepen our understanding of the human immune system and its association with cancer. Our results suggest the putative gene alterations potentially connecting CVID and NHL. Whether these represent a common substrate of CVID phenotype immunodeficiency or rather a risk predisposition for NHL of CVID patients remains unknown and requires prospective studies. These studies, however, may not need to rely on extensive whole-exome sequencing but on customized targeted panels or dPCR approaches leveraging our data, thus facilitating the generation of new evidence in cohorts with a limited number of patients. Beyond conventional cohort studies, the creation of large-scale repositories with comprehensive clinical and immunological annotation (i.e., flow cytometry, immunohistochemistry, RNA-seq immunological signatures) would be a critical step in bridging the elusive gap between immunodeficiency and cancer.

## Data availability statement

The original contributions presented in the study are included in the article/**Supplementary Material**. Further inquiries can be directed to the corresponding author.

## Ethics statement

The studies involving human participants were reviewed and approved by Hospital Clínico San Carlos institutional research Ethics Committee (21/504-E). Written informed consent for participation was not required for this study in accordance with the national legislation and the institutional requirements.

## Author contributions

KG-H, JF-A, RPD, and SS-R contributed to the design of the study and analyzed the data. AO, RPD, PP-S, FS, EN, and MF-A contributed to the analysis of the results and critical review. EF-M contributed to the data collection and designed the tables. KGH and SSR wrote the manuscript. KG-H designed the figures. All authors have read and agreed to the published version of the manuscript.

## Funding

KG-H was supported by The European Social Fund (ESF) through a Río Hortega Grant for Health Research Projects by the Carlos III Health Institute (ISCIII) (CM20/00098). JF-A was supported by a Fundación Sociedad Española de Oncología Médica (SEOM)/EUSA Pharma grant.

## Acknowledgments

We appreciate the insightful and constructive comments made by the reviewers that allowed us to improve the quality of our manuscript.

## Conflict of interest

The authors declare that the research was conducted in the absence of any commercial or financial relationships that could be construed as a potential conflict of interest.

## Publisher’s note

All claims expressed in this article are solely those of the authors and do not necessarily represent those of their affiliated organizations, or those of the publisher, the editors and the reviewers. Any product that may be evaluated in this article, or claim that may be made by its manufacturer, is not guaranteed or endorsed by the publisher.

## Glossary

APDS: activated PI3Kdelta syndrome
APRIL: a proliferating inducing ligand
ATP6AP1: ATPase H+ Transporting Accessory Protein 1
BAFF: B-cell-activating factor of the tumor necrosis factor family
CD19: cluster of differentiation 19
CD20: cluster of differentiation 20
CD21: cluster of differentiation 21
CD81: cluster of differentiation 81
CENTRAL: Cochrane Central Register of Controlled Trials
CMMRD: constitutional mismatch repair deficiency
CVID: common variable immunodeficiency
DOCK8: dedicator of cytokinesis 8
DLBCL: diffuse large B-cell lymphoma
EBV: Epstein–Barr virus
GSEA: gene set enrichment analysis
GPV: germline pathogenic variants
HPV: human papillomavirus
ICOS: inducible T-cell costimulatory
IEI: inborn errors of immunity
IKZF1: IKAROS family zinc finger 1
IRF2BP2: interferon regulatory factor 2-binding protein 2
LRBA: lipopolysaccharide (LPS)-responsive and beige-like anchor protein
MOGS: mannosyl-oligosaccharide glucosidase
NFKB1: nuclear factor kappa B subunit 1
NFKB2: nuclear factor kappa B subunit 2
NGS: next-generation sequencing
NHL: non-Hodgkin’s lymphoma
OR: odds ratio
PID: primary immunodeficiency
PIK3CD: phosphatidylinositol-4,5-bisphosphate 3-kinase catalytic subunit delta
PIK3R1: phosphoinositide-3-kinase regulatory subunit 1
PRISMA: Preferred Reporting Items for Systematic Reviews and Meta-Analyses
PRKCD: protein kinase C delta
PTEN: phosphatase and tensin homolog
SHM: somatic hypermutation
STAT3: signal transducer and activator of transcription 3
STXBP2: syntaxin-binding protein 2
TACI: transmembrane activator and CAML interaction
TCGA: The Cancer Genome Atlas
TRNT1: TRNA nucleotidyl transferase 1
TNFSF12: TNF-related weak inducer of apoptosis (TWEAK), TNF superfamily member 12
