# Supplementary material for: Genomic crossroads between non-Hodgkin’s lymphoma and common variable immunodeficiency
Source: Front Immunol. 2022 Aug 5;13:937872. doi: 10.3389/fimmu.2022.937872 (PMC9390007; doi:10.3389/fimmu.2022.937872)
Supplement: Supplementary file 1 [file DataSheet_1.docx]

**Supplementary Data**


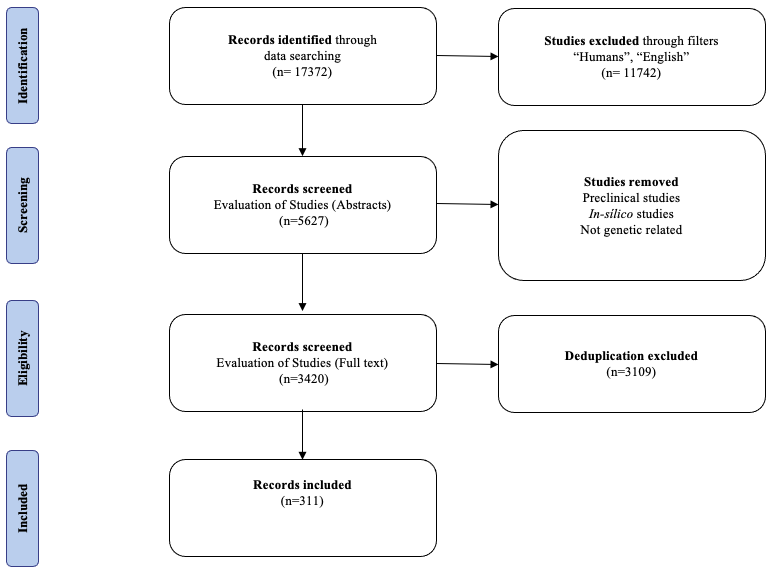


**Supplementary Figure 1.** Flow Chart that identifies the successive steps and filters used for the recruitment of the studies included in the meta-analysis. Preferred Reporting Items for Systematic Reviews and Meta-analyses (PRISMA).

**Supplemental Figure 2. PIK3R1 as a central node for signaling pathway interaction in CVID. A.** Top 3 representation of the ranking gene common for NHL and CVID. No significant direct interaction was observed between *PIK3CD, KMT2C* and *STAT3* in a first degree of separation (*PIK3CD/STAT3* 2 degrees separation*, p-value: 0.013; PIK3CD/KMT2C* 3 degrees separation*, p-value: 0.636)*^70^. **B.** A level of interaction of TOP3, a common point is evidenced between the genes described due to the association of *PIK3R1* in their common signaling pathways. *PIK3R1* represents the 7th place in the gene ranking. Interaction score: Highest confidence (0.9) for *STAT3* and *PIK3CD* with *PIK3R1*; Medium confidence (0.4) for *KMT2C* and *PIK3R1*. PPI enrichment p-value: 0.0491. **C.** Top 6 representation of the ranking gene common for NHL and CVID. Medium confidence (0.4), p-value: 0.0397. **D.** A higher level of interaction of TOP 7, a new common point is evidenced between the genes described due to the association of *PIK3R1* in their common signaling pathways. Interaction score: Highest confidence (0.9) for *STAT3*, PTEN and *PIK3CD* with *PIK3R1*; Medium confidence (0.4) for *KMT2C* and *PIK3R1*. Low Confidence (0.15) for *NFKB2* and MSH2 with PIK3R1; PPI enrichment p-value: 0.0265.

(**Supplementary Table 1 and 2** Attached in supplementary file xlsx)

**Supplementary Table 1.** Total of 50 genes mainly CVID phenotype-associated. Pathogenic variants, effect as CVID phenotype, characteristics of the mutation, inheritance and clinical profile described in the literature.

**Supplementary Table 2.** List of the main germline CVID-associated pathogenic variants found as somatic variants in the NHL databases.

| **Sources for gene sets** |  |
| --- | --- |
| **Pathway Collection** | **Original Pathway Source** |
| Biocarta | <http://www.biocarta.com> |
| cBioPortal for Cancer Genomics | <https://www.cbioportal.org/> |
| Clinvar-NCBI | <https://www.ncbi.nlm.nih.gov/clinvar/> |
| EnrichR | <https://maayanlab.cloud/Enrichr/> |
| Reactome | <http://www.reactome.org/> |
| Gene Ontology | <http://www.geneontology.org/> |
| STRING Database | <https://string-db.org/> |
| The Human Gene Connectome Server of Rockefeller University | <https://hgc.rockefeller.edu/> |

**Supplementary Table 3.** Sources for gene sets.
